# Experimental and Computational Analysis of *Para*-Hydroxy Methylcinnamate following Photoexcitation

**DOI:** 10.3390/molecules26247621

**Published:** 2021-12-15

**Authors:** Jack Dalton, Gareth W. Richings, Jack M. Woolley, Temitope T. Abiola, Scott Habershon, Vasilios G. Stavros

**Affiliations:** Department of Chemistry, University of Warwick, Coventry CV4 7AL, UK; J.Dalton.1@warwick.ac.uk (J.D.); G.Richings@warwick.ac.uk (G.W.R.); Jack.Woolley@warwick.ac.uk (J.M.W.); Temitope.Abiola@warwick.ac.uk (T.T.A.)

**Keywords:** ultrafast spectroscopy, DFT, gas-phase, VMI, photochemistry, photophysics, cinnamate

## Abstract

*Para*-hydroxy methylcinnamate is part of the cinnamate family of molecules. Experimental and computational studies have suggested conflicting non-radiative decay routes after photoexcitation to its S_1_(ππ*) state. One non-radiative decay route involves intersystem crossing mediated by an optically dark singlet state, whilst the other involves direct intersystem crossing to a triplet state. Furthermore, irrespective of the decay mechanism, the lifetime of the initially populated S_1_(ππ*) state is yet to be accurately measured. In this study, we use time-resolved ion-yield and photoelectron spectroscopies to precisely determine the S_1_(ππ*) lifetime for the s-*cis* conformer of *para*-hydroxy methylcinnamate, combined with time-dependent density functional theory to determine the major non-radiative decay route. We find the S_1_(ππ*) state lifetime of s-*cis para*-hydroxy methylcinnamate to be ∼2.5 picoseconds, and the major non-radiative decay route to follow the [^1^ππ*→^1^nπ*→^3^ππ*→S_0_] pathway. These results also concur with previous photodynamical studies on structurally similar molecules, such as *para*-coumaric acid and methylcinnamate.

## 1. Introduction

Cinnamates and coumaric acids have been widely studied to elucidate their remarkable light absorbing properties, driven by efficient non-radiative decay (NRD) pathways [1,2,3,4,5]. In nature, these chromophores are embedded in proteins to enable vitally important processes to occur. A prime example is phototaxis in the photoactive yellow protein (PYP). PYP was first discovered in the *Ectothiorhodospira halophila* bacterium [6,7,8] and utilizes the photoisomerization of *para*-coumaric acid (*p*-CA) to facilitate a negative phototactic response towards blue light, thus protecting the bacterium from potentially harmful photodamage [9,10,11,12]. Similar families of molecules are used in sunscreen formulations due to their photoprotective properties against potentially harmful ultraviolet A (UVA, 315–400 nm) and B (UVB, 280–315 nm) radiation from the Sun [13,14,15].

**Figure 1:**
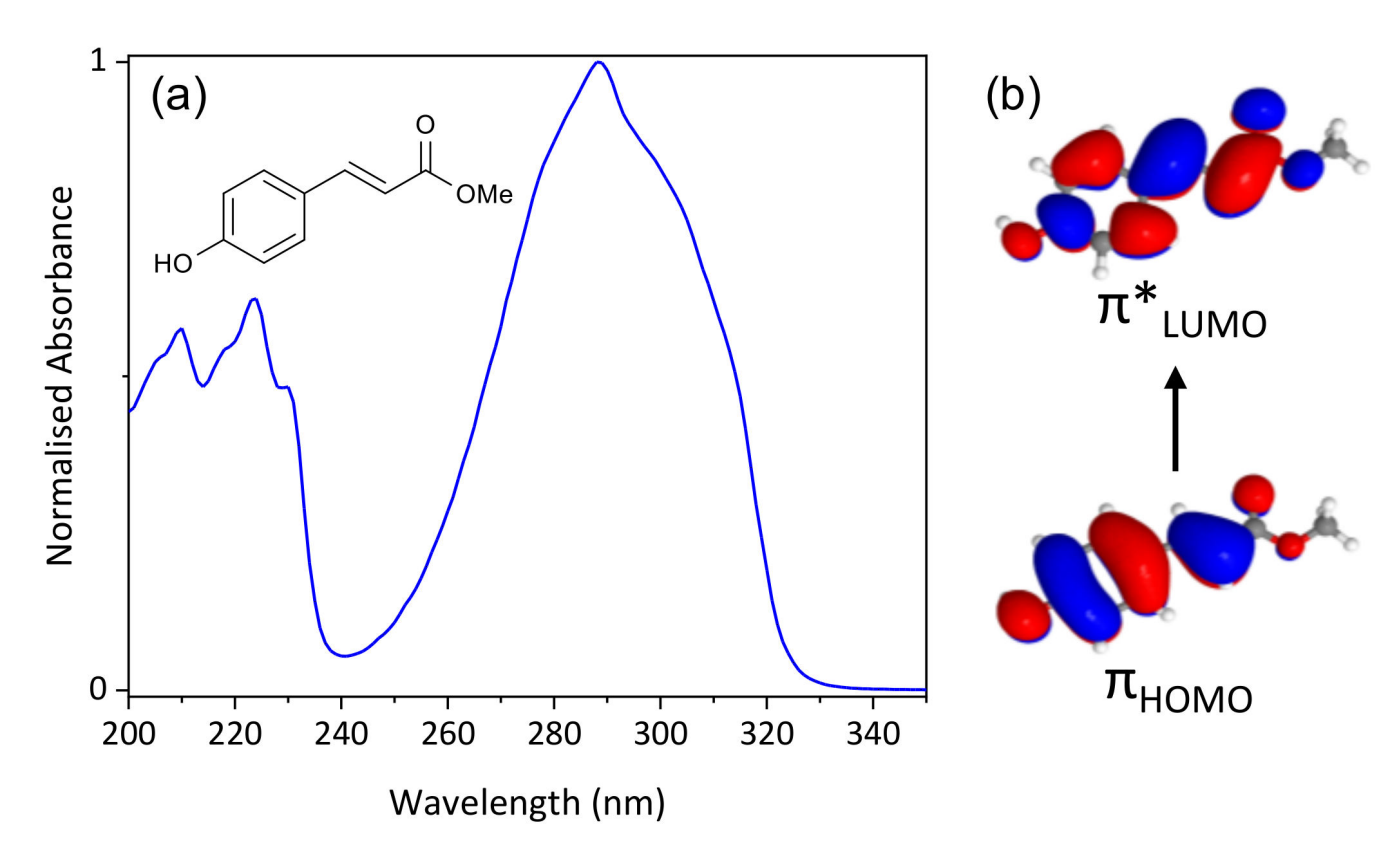
(**a**) UV absorption spectrum of *para*-hydroxy methylcinnamate (*p*-HMC) (structure shown inset) in cyclohexane. (**b**) *p*-HMC’s π highest occupied molecular orbital (π_HOMO_) and π* lowest unoccupied molecular orbital (π*_LUMO_) relating to the S_1_(ππ*) state.

*Para*-hydroxy methylcinnamate (*p*-HMC; Figure 1a) is the simplest ester derivative of *p*-CA and exhibits a strong absorption peak in the UVB region of the electromagnetic spectrum. Upon UV irradiation, the optically bright S_1_(ππ*) state is predominantly populated and undergoes relaxation via multiple NRD processes, returning to the electronic ground S_0_ state. Although the decay dynamics of *p*-HMC following photoexcitation to the ^1^ππ* state are well-studied, there have been some conflicting conclusions regarding the dominant NRD route in the gas-phase. An early theory suggested that decay via the ^1^πσ* dissociative state may dominate, analogous to what is observed in phenol [16]. The lowest lying ^1^πσ* state in phenol is dissociative along the O–H bond and ultimately leads to a conical intersection (CI) with the S_0_ state, providing rapid relaxation of the molecule [17,18]. Upon deuteration of the OH moiety, a dramatic change in excited-state lifetime is observed, indicating that the excited-state decay in phenol is mediated via tunnelling [19,20]. In contrast, deuteration of the OH moiety in *p*-HMC results in very little change in excited-state lifetime, implying that O–H fission is unavailable to *p*-HMC following photoexcitation to the S_1_ state [21].

Following this observation, Smolarek et al. proposed an alternative NRD route for *p*-HMC, mirroring that of *p*-CA. This was based on the similarities in linewidths (and hence excited state lifetimes) of the photoexcitation spectra of *p*-HMC and *p*-CA, implying a comparable NRD route in both systems [21,22].

**Figure 2:**
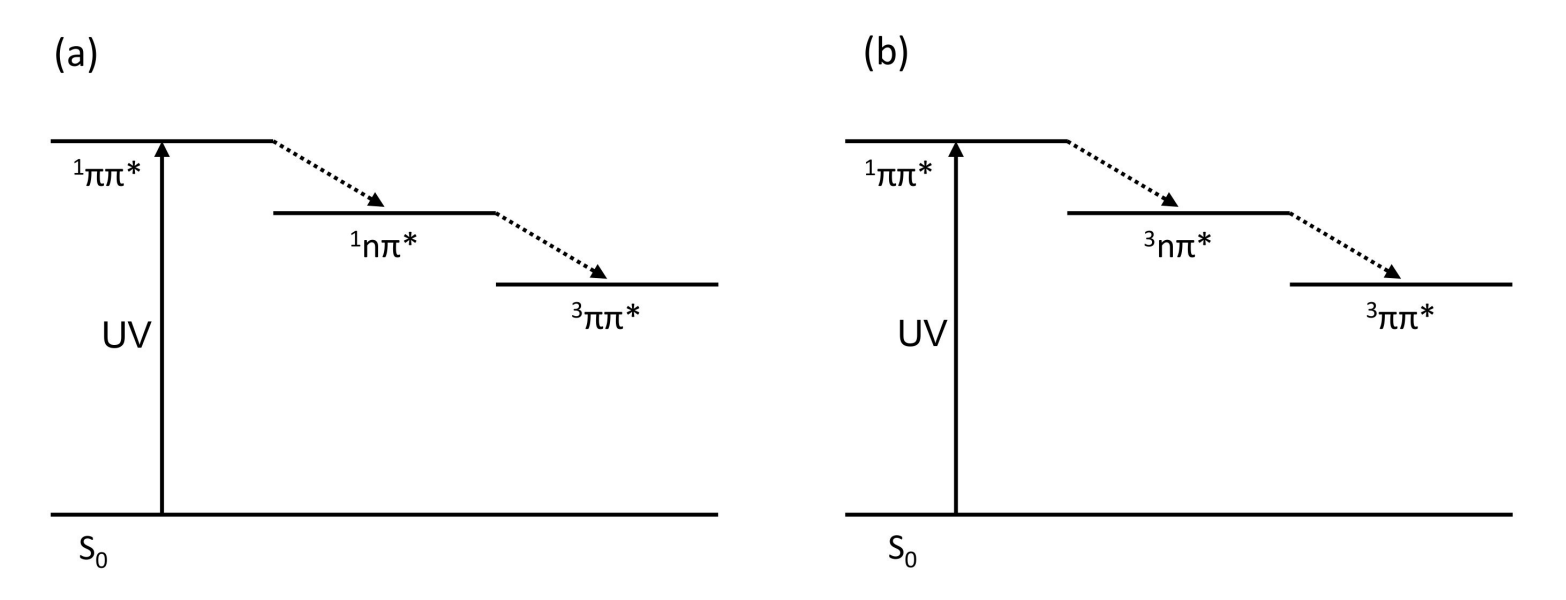
Possible non-radiative decay (NRD) routes following the absorption of UV light. (**a**) Internal conversion from the ^1^ππ* to the ^1^nπ* state followed by intersystem crossing to the ^3^ππ* state. (**b**) Direct intersystem crossing from the ^1^ππ* to the ^3^nπ* state followed by internal conversion to the ^3^ππ* state.

*p*-CA and methylcinnamate (MC), both structurally very similar to *p*-HMC, are photo-chemically and photo-physically well understood. It therefore stands to reason that they can be used as starting models for unravelling the NRD dynamics of *p*-HMC. Upon photoexcitation of *p*-CA to the ^1^ππ* state in the gas-phase, the NRD process shown in Figure 2a dominates, with the observed lifetime of the ^1^ππ* and ^3^ππ* states measured as ≤10 picoseconds (ps) and 20 ± 4 nanoseconds (ns), respectively [5]. For MC, the same NRD process dominates with the ^1^ππ*, ^1^nπ* and ^3^ππ* states having been experimentally observed. The observed lifetimes are also remarkably similar to *p*-CA, being 4.5 ± 1.1 ps and 20 ns for the ^1^ππ* and ^3^ππ* states, respectively [23,24].

In contrast, a study by Yamazaki et al. for *para*-methoxy methylcinnamate (*p*-MMC), a close structural derivative of *p*-HMC, suggests that multistep intersystem crossing (ISC) from the ^1^ππ* state to the ^3^ππ* state could involve an intermediate ^3^nπ* state [25]. Similarly, a recent computational study by Kinoshita et al. suggests a major NRD route for *p*-HMC and *p*-MMC, based on MC, that supports the involvement of the aforementioned intermediate ^3^nπ* state [1]. Their rationale is based on the fact that OH and OMe are π donors; the highest occupied molecular orbital (π) is destabilised by these π-donors, reducing the π-π* energy gap and consequently decreasing the energy of the ππ* states while having minimal effect on the nπ* states. This relative decrease in energy of the ππ* states reverses the adiabatic ordering of the ^1^ππ* and ^1^nπ* states, opening new major NRD routes. For *p*-HMC and *p*-MMC, Kinoshita et al. suggest the major NRD shown in Figure 2b.

**Scheme 1:**
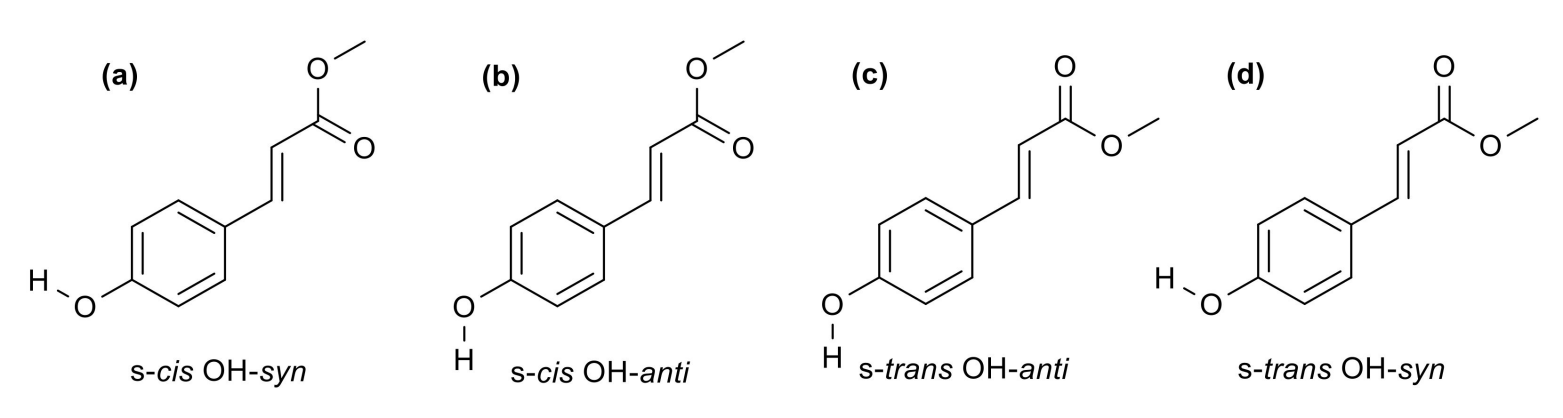
Molecular structures for the four conformers of *p*-HMC: (**a**) s-*cis* OH-*syn*, (**b**) s-*cis* OH-*anti*, (**c**) s-*trans* OH-*anti*, and (**d**) s-*trans* OH-*syn*.

There are four conformers of *p*-HMC, shown in Scheme 1. The S_1_←S_0_ origin bands of these four conformers are located at 32,711.0, 32,715.9, 32,879.5, and 32,887.7 cm^−1^ for, respectively, (a), (b), (c) and (d) [26]. From the same study, Tan et al. used the linewidths from UV-UV depletion spectroscopy to propose lower lifetime limits of 1.5 and 2.5 ps for the s-*cis* and s-*trans* conformers, respectively. Since this work, Kinoshita et al. and Shimada et al. have carried out time-resolved pump-probe studies on *p*-HMC. In both of these studies, the ^1^ππ* lifetime was inferred to be ≤9 ps [27,28]. The ^3^ππ* state has also been experimentally observed at 19,020 cm^−1^ and shown to have an average lifetime of 26 ± 4 ns [28]. However, the optically dark intermediate ^1^nπ* or ^3^nπ* state is lacking a direct observation, and therefore opens speculation for the major NRD route. This lack of observation may be attributed to weak nπ*←S_0_ absorption that is swamped by the strong S_1_(ππ*)←S_0_ absorption in combination with a large ionisation energy to remove an electron from an nπ* state [28].

In the present study, our aim is to: (a) precisely determine the gas-phase lifetime of the S_1_(ππ*) state in *p*-HMC; and (b) contribute towards determining the major NRD route from the S_1_(ππ*) state. To address (a), we use femtosecond (fs) to ps time-resolved ion-yield (TR-IY) and time-resolved photoelectron spectroscopy (TR-PES). The approximate 200 cm^−1^ energy difference in the origin bands of the s-*cis* and s-*trans* conformers allows us to selectively excite and precisely measure the S_1_(ππ*) state lifetime of the s-*cis p*-HMC conformer. To address (b), time-dependent density functional theory (TDDFT) is used to calculate the possible relaxation pathways originating from the S_1_(ππ*) state in the s-*cis* conformer of *p*-HMC.

## 2. Results and Discussion

### 2.1. Experimental

**Figure 3:**
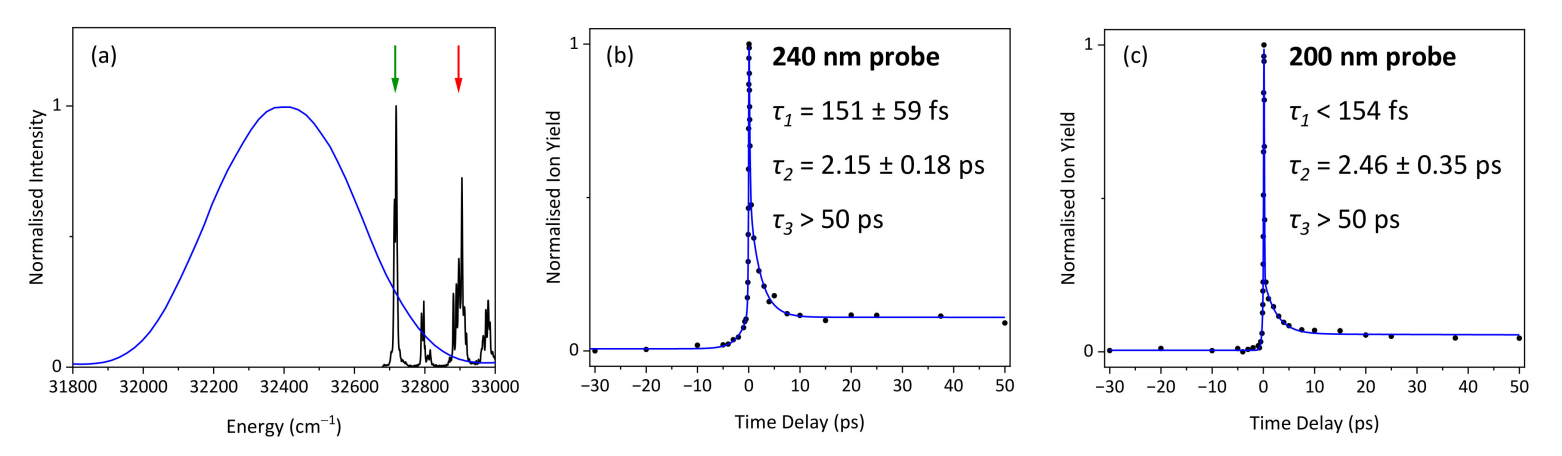
(**a**) Resonance enhanced multiphoton ionisation spectrum of *p*-HMC, relative to the S_1_(ππ*) state, provided by Tan et al. [26]. The green arrow indicates the s-*cis* conformer and the red arrow indicates the s-*trans* conformer. Overlaid is the 308.5 nm (32,415 cm^−1^) pump laser spectral profile which is used to selectively excite (predominantly) s-*cis p*-HMC to the S_1_(ππ*) state. (**b**) Time-resolved ion-yield (TR-IY) transient of *p*-HMC at 308.5 nm pump and 240 nm probe with a 3 bar helium backing pressure. (**c**) TR-IY transient of *p*-HMC at 308.5 nm pump and 200 nm probe with a 1.5 bar helium backing pressure to reduce cluster formation (discussed *infra*). In (**b**,**c**), the polarisations of the pump and probe beams were parallel with respect to each other, and the negative dynamics were zeroed as the baseline fluctuated around zero due to poor background subtraction.

**Figure 4:**
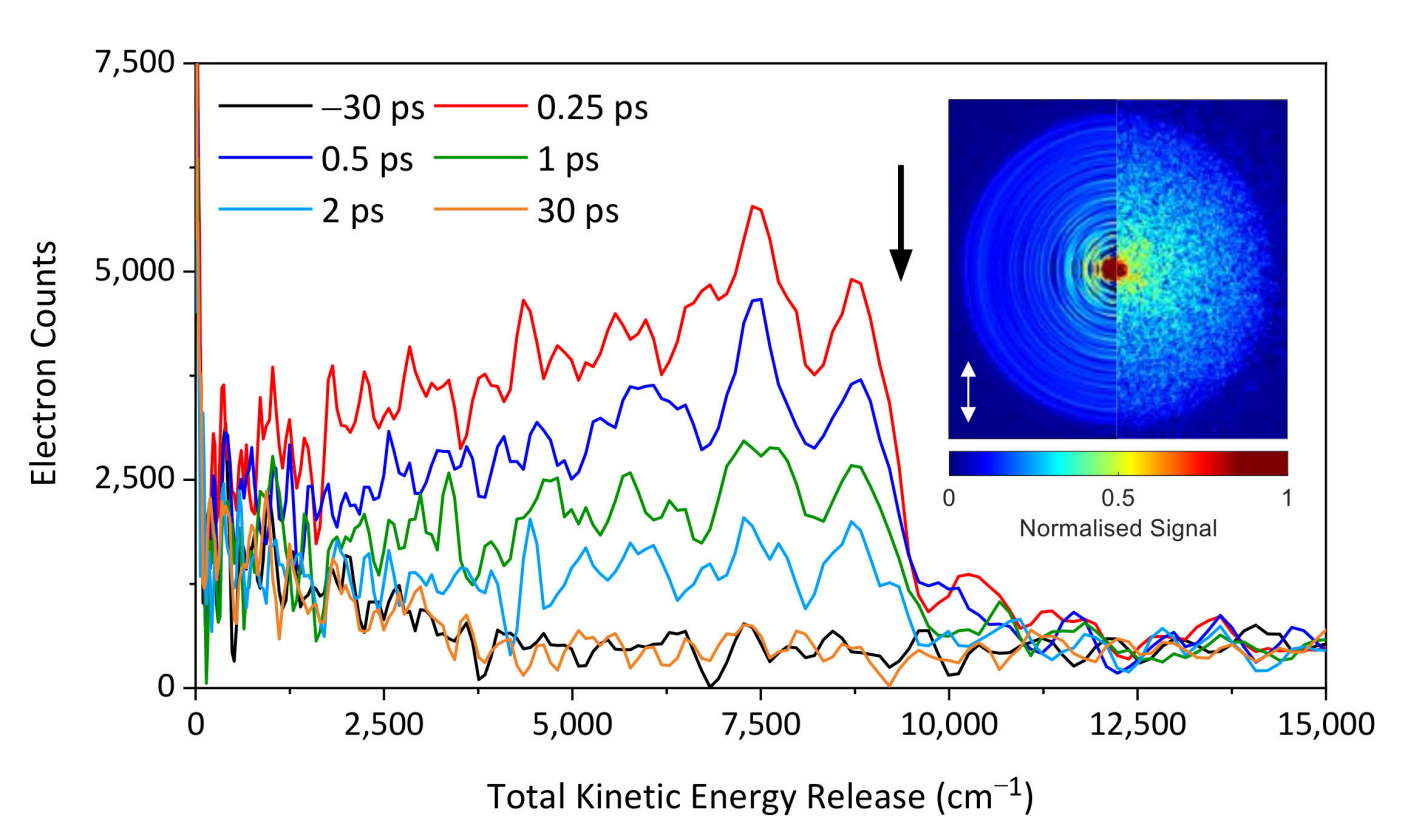
Total kinetic energy release (TKER) spectra of *p*-HMC obtained using the 308.5 nm pump and 240 nm probe with a 1.5 bar helium backing pressure. The following pump-probe time delays are used: Δ*t* = −30, 0.25, 0.5, 1, 2 and 30 ps and an expected *TKER_max_* of around 9360 cm^−1^ is indicated by the black arrow. Inset: right half presents the recorded image, while the left half presents the reconstructed slice through the centre of the original three-dimensional (3D) ion distribution at Δ*t* = 0.25 ps (the white arrow indicating the electric field polarisation of the laser pulses). The polarisations of the pump and probe beams were parallel with respect to each other.

Figure 3b,c show TR-IY transients for the s-*cis* conformer of *p*-HMC including the associated time constants and errors for the forward dynamics (pump-probe time delay (Δ*t*) > 0), see Section 3.1. For these transients, the pump beam was centred at 308.5 nm (32,415 cm^−1^) to ensure s-*cis* (*syn* and *anti*) conformer-specific excitation of *p*-HMC to the S_1_(ππ*) state [26,27,28]. The spectral overlap of the pump beam with the S_1_(ππ*) state of relevant conformers can be seen in Figure 3a. For the TR-IY transient in Figure 3b, the probe beam was centred at 240 nm (*p*-HMC’s minimum absorption, Figure 1a) to minimise both the probe-only ion-signal and reverse dynamics (Δ*t* < 0). The ionisation potential (IP) and the energy of the ^3^ππ* state are located around 65,020 and 19,020 cm^−1^, respectively [27,28]. As a result, a probe beam at 240 nm (41,667 cm^−1^) ensures that ionisation is only optically accessible from the S_1_(ππ*) state (according to the proposed NRD paths shown in Figure 2), since the first two ionisation levels are of π^−1^ character and there is insufficient energy to reach the D_2_(n^−1^) ionisation state from any possible nπ* states [28]. This is evidenced by the total kinetic energy release (TKER) spectra in Figure 4 (discussed below), derived from reconstructed photoelectron images (Figure 4 inset), which shows no real structural change upon varying Δ*t*, apart from a fast (<5 ps) decay. Knowing this, we can reasonably assume that the excited state lifetimes extracted from the TR-IY transient using a 240 nm probe in Figure 3b are directly related to the S_1_(ππ*) state lifetime. For completeness, the probe beam in Figure 3c is centred at 200 nm (50,000 cm^−1^) to enable ionisation from the S_1_(ππ*) state, any possible intermediate states, and the final ^3^ππ* excited state located 46,000 cm^−1^ below the IP. Since the ^3^ππ* state is known to have an average lifetime of 26 ± 4 ns, Figure 3c is expected to include an additional long-lived lifetime component (assuming it is formed within the time-window of our experiment, 900 ps) that would not be seen with the 240 nm probe due to the insufficient energy required to ionise from that state [28]. However, both transients exhibit the same dynamics, with three extracted time-constants *τ*_1–3_ using the procedure outlined in Section 3.1.

To start our discussion, we will address the first time constant, *τ*_1_ (∼150 fs). This lifetime is much shorter than the lower lifetime limit of 1.5 ps suggested by Tan et al. from the depletion spectra linewidth of the S_1_(ππ*) state in s-*cis p*-HMC, and is therefore likely not the lifetime of the S_1_(ππ*) state [26]. As mentioned *supra*, when using a 240 nm probe, the S_1_(ππ*) is the only state available for ionisation. Knowing this, we assign *τ*_1_ to initial evolution out of the Franck–Condon (FC) region, combined with any initial intramolecular vibrational redistribution, in keeping with previous studies [3,29,30,31].

The second time constant, *τ*_2_, from the TR-IY transients in Figure 3b,c is 2.15 ± 0.18 ps and 2.46 ± 0.35 ps, for a 240 nm and 200 nm probe, respectively. These lifetimes broadly agree with the lower limit of 1.5 ps suggested by Tan et al. and the ≤9 ps suggested by Kinoshita et al. for the lifetime of the S_1_(ππ*) state in s-*cis p*-HMC [26,28]. Additionally, TR-PES (with a 240 nm probe) derived TKER spectra, shown in Figure 4, almost returns to baseline by 2 ps with no real structural change or shift in the maximum TKER (*TKER_max_*). This implies that the ps decay is solely related to the decay of the initially populated excited S_1_(ππ*) state. The *TKER_max_* is also in agreement with the expected *TKER_max_* for *p*-HMC, originating from excitation to the origin of s-*cis p*-HMC. Using Equation (1), the expected *TKER_max_* can be calculated:

(1) $$TKER_{max}=E_{OB}+E_{probe}-IP$$

where *E_OB_* is the energy of the S_1_(ππ*) origin band for the s-*cis* conformers, *E_probe_* is the energy of the probe (41,667 cm^−1^), and *IP* is the ionisation potential (65,020 cm^−1^). As a result, the expected *TKER_max_* is 9358 cm^−1^ and 9362.9 cm^−1^ for the two s-*cis* conformers, indicated by the black arrow in Figure 4 [26,27]. Encouragingly, this appears in excellent agreement with the observed *TKER_max_* from the TKER spectra shown in Figure 4. Considering these results and their agreement with previous results, *τ*_2_ is assigned to the lifetime of the S_1_(ππ*) state of *p*-HMC in its s-*cis* configuration.

**Figure 5:**
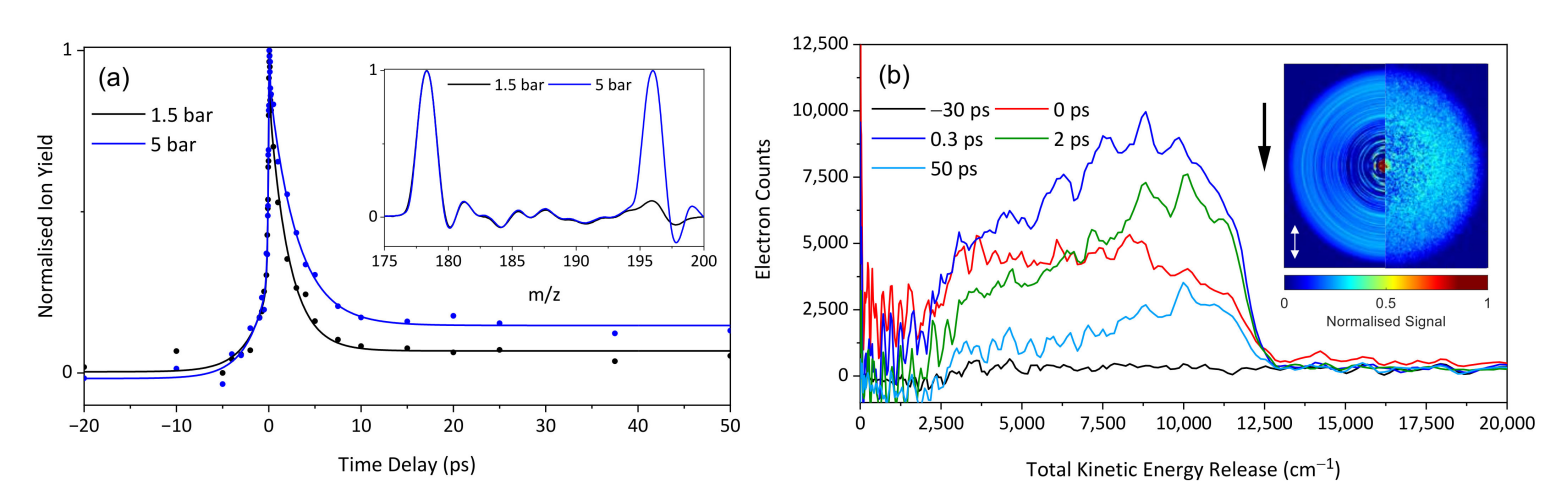
(**a**) TR-IY transient of *p*-HMC at 308.5 nm pump and 240 nm probe with a 1.5 and 5 bar helium backing pressure showing a larger baseline offset at 5 bar. Inset: time-of-flight mass spectrum, taken at 1.5 and 5 bar, showing the majority of ion-signal is *p*-HMC^+^ (m/z = 178) at 1.5 bar and at 5 bar, ion-signal corresponding to [*p*-HMC + H_2_O]^+^ (m/z = 196) additionally appears. (**b**) TKER spectra of *p*-HMC obtained using the 308.5 nm pump and 240 nm probe with a 5 bar helium backing pressure. The following pump-probe time delays are used: Δ*t* = −30, 0, 0.3, 2 and 50 ps and an expected *TKER_max_* of 12,497 cm^−1^ is indicated by the black arrow. Inset: right half presents the recorded image, while the left half presents the reconstructed slice through the centre of the original 3D ion distribution at Δ*t* = 0.3 ps (the white arrow indicating the electric field polarisation of the laser pulses). For all data, the polarisations of the pump and probe beams were parallel with respect to each other.

Finally, the third time constant, *τ*_3_, is long-lived in both 240 nm probe and 200 nm probe transients and extends beyond the maximum time-window of our measurements, 900 ps (ESI, Figures S1 and S2). When probing at 240 nm, this result is surprising, since the only optically ionisable state is the S_1_(ππ*). As such, this merited further investigation. The relative amplitude of this feature is dependent on the helium backing pressure, which can be seen in Figure 5a for the TR-IY transients at 1.5 and 5 bar helium. An increase in the backing pressure increases collisional cooling, which leads to formation of water clusters, shown in Figure 5a inset. At 1.5 bar helium, most of the ion-signal is attributed to *p*-HMC, but at 5 bar helium, the data includes a large ion-signal that is assigned to *p*-HMC + H_2_O clusters. Furthermore, TKER spectra taken at 5 bar helium (Figure 5b), derived from reconstructed photoelectron images (Figure 5b inset), are clearly different to those at 1.5 bar helium (Figure 4). The first notable difference is the increase in *TKER_max_* from approximately 9360 cm^−1^ to >12,000 cm^−1^. The *TKER_max_* in Figure 5b accords with the expected *TKER_max_*, 12,497 cm^−1^, when using an IP of 61,585 cm^−1^, determined by Smolarek et al., for a *p*-HMC + H_2_O cluster (due to non-selective excitation, the expected *TKER_max_* is obtained using the pump energy and not the energy of the S_1_(ππ*) origin band for *p*-HMC + H_2_O) [21]. From this, we can confirm the presence of water clusters at higher backing pressures. Additionally, it is clearly evident that the TKER spectra obtained at 5 bar helium do not tend to zero (*cf.* at 1.5 bar helium); there is still population of the first excited state at 50 ps. This correlates strongly with the long-lived S_1_(ππ*) state lifetime for the *p*-HMC + H_2_O cluster, previously measured by ps pump-probe spectroscopy [27]. Considering the above, the marked baseline offset in the TR-IY transients (notably with a 240 nm probe) described by the long-lived component *τ*_3_, is attributed to the S_1_(ππ*) state in *p*-HMC + H_2_O clusters. 

We end this section with a caveat: When probing at 200 nm, the mild baseline offset is expected since we can, potentially, ionise from both the ^1^nπ* and ^3^ππ* states. Consequently, we are unable to determine whether *τ*_3_ from the 200 nm probe TR-IY transient (see Figure 3c) is a result of cluster dynamics, ionisation from an nπ* intermediate state, a ^3^ππ* state, or any of these combinations.

### 2.2. Computational

As the experiments were carried out on the s-*cis* conformers of *p*-HMC, we have carried out calculations using both OH-*syn* and OH-*anti* conformations (Scheme 1a,b), respectively); for brevity we will refer to these as *syn* and *anti* in what follows.

Initially the ground state geometries of the two conformers were optimised using density functional theory (DFT) and the excited state energies evaluated at both points. It was found that the *anti* conformation had the lower S_0_ energy at the minimum by 38 cm^−1^. As such, all energies reported here are given relative to this minimum, whichever conformation they are. We are interested in the NRD mechanism of this molecule, specifically whether the ^1^nπ* or ^3^nπ* state plays an intermediate role in that decay, and, as such, we note that, in both conformations, the ^1^nπ* is the ninth lowest energy excited state (see data in ESI, Tables S1 and S2). There are five triplets below the bright S_1_/^1^ππ* state, followed by a sixth triplet before two more singlets, S_2_ being a less bright (by a factor of over 4) ^1^ππ* state and S_3_ being the dark ^1^nπ* of interest (the non-bonding orbital contains the lone pair on the carbonyl oxygen, see Scheme 1). Above these two singlets is another triplet state, which is the limit of our interest. We note that T_5_ in both conformations is of nπ* character, whilst all other triplets have ππ* character. It is thus clear that excitation will be predominantly to the S_1_ state, that being by far the brightest in both cases. The vertical excitation energies are 39,993 cm^−1^ (*anti*) and 39,844 cm^−1^ (*syn*), meaning that, whilst the absolute values of the excitations are about 7000 cm^−1^ above the experimental values (which are adiabatic, so not directly comparable), they are close in energy with the correct ordering. In both cases, we also note that there is less than 3000 cm^−1^ between all three singlet excited states.

**Figure 6:**
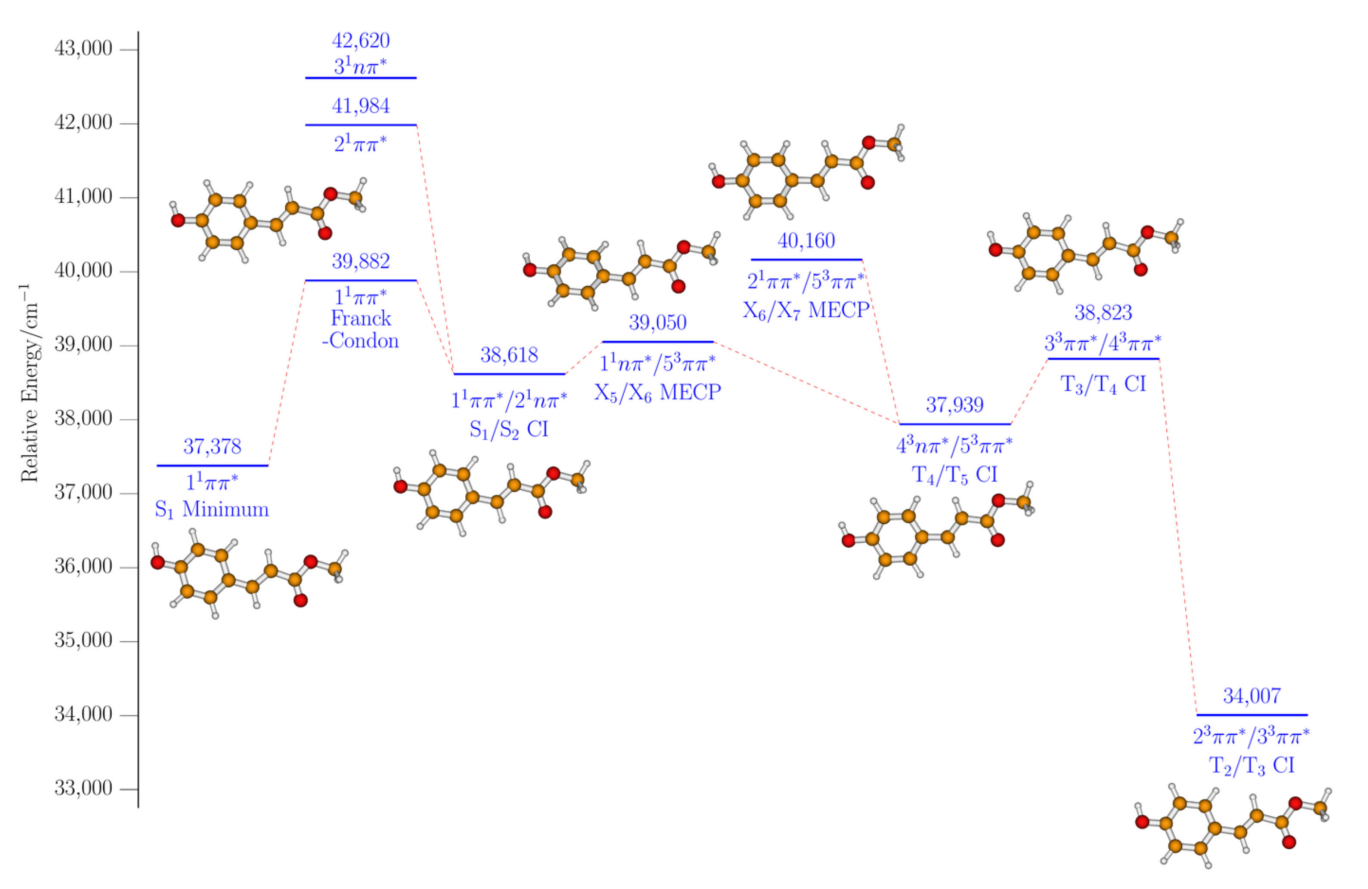
Energy landscape showing points relevant to the NRD of p-HMC. Geometries were optimised starting in the syn conformation. All energies are relative to the S0 state of the anti conformer Franck-Condon (FC) point. Energies, in cm−1, are given above the blue lines; immediately below are the states in question; and below that are the labels of the optimised points. We have also included images of the molecular structures of the optimised points, above or below the lines for clarity. From left to right are the data for the S1 minimum; the FC point; the S1/S2 conical intersection (CI); the X5/X6 (S1/T5) minimum energy crossing point (MECP); the X6/X7 (S2/T5) MECP; the T4/T5 CI; the T3/T4 CI; and the T2/T3 CI.

**Figure 7:**
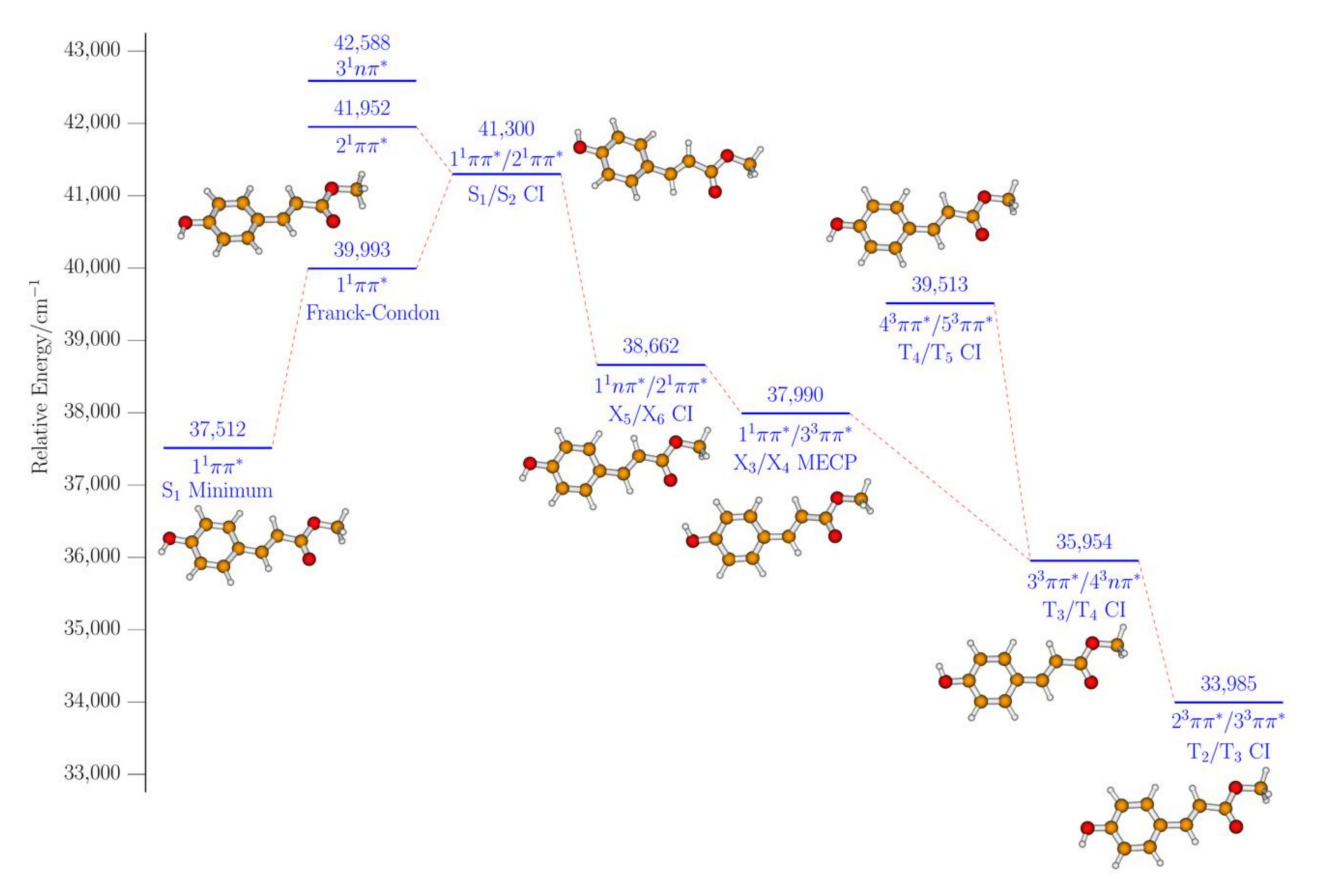
Energy landscape showing points relevant to the NRD of *p*-HMC. Geometries were optimised starting in the *anti* conformation. All energies are relative to the S_0_ state of the *anti* conformer FC point. Energies, in cm^−1^, are given above the blue lines; immediately below are the states in question; and below that are the labels of the optimised points. We have also included images of the molecular structures of the optimised points, above or below the lines for clarity. From left to right are the data for the S_1_ minimum; the FC point; the S_1_/S_2_ CI; the X_5_/X_6_ (S_1_/S_2_) CI; the X_3_/X_4_ (S_1_/T_3_) MECP; the T_4_/T_5_ CI; the T_3_/T_4_ CI; and the T_2_/T_3_ CI.

Given the two optimised, FC geometries, they were then used as starting points for optimisation on the excited states: minimum energy geometries on all ten excited states were sought (using restricted Kohn–Sham (RKS)), as were the CIs between all energy adjacent states of the same multiplicity (using RKS), and finally all crossings between energy adjacent states, regardless of multiplicity (using unrestricted Kohn–Sham (UKS), which does not distinguish states based on multiplicity in ORCA). We note that not all optimisations were successful, with some minima not being located in spite of restarted calculations at the most recently visited geometry. All data is included in Tables S1–S5 in the ESI (where the data is presented in energy order and labelled S*_x_* and T*_y_* for clarity, because the character of the states change at different geometries), which gives energies relative to the *anti* S_0_ minimum, but the main data to be discussed in what follows is shown in Figure 6 and Figure 7. Here, Figure 6 contains information about geometries optimised from the *syn* conformer and Figure 7 contains information about points found starting with the *anti* conformer. We will focus our discussion on these selected points that best relate to the NRD of s-*cis p*-HMC. With these selected points, we have also tried to locate the transition states (TSs) between relevant pairs of conformers in order to assess any energy barriers between them. To note, there appears to be no energy barrier from the *anti* FC to the *syn* FC point. Therefore, initial excitation to the *anti* conformer could be followed by rotation of the OH group to the *syn* conformer, prior to any excited state crossings.

Looking at the deactivation of *p*-HMC by crossing to lower energy states, there are two proposed mechanisms, both of which involve ISC to the triplet manifold through which deactivation to S_0_ occurs; the difference between the two is whether the first step is internal conversion to a ^1^nπ* state before the ISC step or whether the excited molecule goes from ^1^ππ* to ^3^ππ* via ^3^nπ*. To assess this, we first look at the ISC process to see what feasible paths there are by examining the spin-orbit couplings (SOCs) between the singlet and triplet states at the minimum energy crossing points (MECPs) found using UKS TDDFT. A total of ten singlet–triplet MECPs were found, four optimisations starting in the *anti* conformer and six in the *syn*. In what follows, we will denote the MECPs found by the initial conformer (*anti* or *syn*) and the crossed states, so *anti*-1^1^ππ*/3^3^ππ* is the crossing of 1^1^ππ* and 3^3^ππ* starting from the *anti*-FC point. The optimised *anti*-1^1^ππ*/3^3^ππ* geometry finished as a *syn* conformer, whilst all other nine systems stayed in their original *anti* or *syn* conformation. The *anti*-1^1^ππ*/3^3^ππ* MECP geometry is non-planar with carbonyl C (Scheme 1) having a dihedral angle of 165° with the ring. The state energies at this MECP are similar to those found at *syn*-1^1^ππ*/3^3^ππ* point, being only a few hundred cm^−1^ different in all cases. The *syn*-1^1^ππ*/3^3^ππ* geometry has a dihedral angle of −178.5° between the carbonyl C and the ring, so the geometries of the MECPs are different, suggesting they lie on different seams of intersection.

Looking at the SOCs between the singlet and triplet states, shown in Table 1, the only couplings with a magnitude of greater than 1 cm^−1^ are at the *anti*-1^1^ππ*/3^3^ππ* crossing (1.27 cm^−1^), the *syn*-2^1^ππ*/5^3^ππ* MECP (4.71 cm^−1^) and the *syn*-1^1^nπ*/5^3^ππ* geometry (11.25 cm^−1^). As already noted, the *anti*-1^1^ππ*/3^3^ππ* geometry is actually *syn*-like. We would expect the greatest rates of ISC to occur at these three crossing points, so let us consider the characters of the states there in more detail. At the *anti*-1^1^ππ*/3^3^ππ* MECP, both intersecting states have ππ* character. At the *syn*-1^1^nπ*/5^3^ππ* MECP, the singlet is over 84% nπ* with a little ππ* mixed in, whilst at *syn*-2^1^ππ*/5^3^ππ*, the singlet is predominantly ππ*, but with over 20% nπ* character included. From this analysis, we conclude that the largest SOCs at the MECPs are between singlets with significant nπ* components in their character (the more the better) and ππ* triplets. This is in accordance with El-Sayed’s rule and therefore, unsurprising [32]. Pictures of the orbitals involved in the excitations, with largest components, which characterise the states that cross at the two geometries with largest SOCs are given in Figure S6 of the ESI. Even the *anti*-1^1^ππ*/3^3^ππ* MECP noted here has a SOC an order of magnitude lower than that at the *syn*-1^1^nπ*/5^3^ππ* geometry. It thus appears that the rate of ISC will be greater going from a ^1^nπ* state to a ^3^ππ* state in the triplet manifold, rather than from the ^1^ππ* state, suggesting that the NRD of *p*-HMC is mediated by the ^1^nπ* state. In order for this to occur, there must be a route from the initially excited ^1^ππ*state to the ^1^nπ*, i.e., we need a CI and also a path to the crossing points from the FC point with a low barrier or none at all. We note that none of the other MECPs between singlet and triplet states, as listed in Table 1, involved the ^3^nπ* state, meaning that we have not located a path from the optically bright ^1^ππ* state to the ^3^nπ* state, which was the alternative NRD pathway proposed (Figure 2b). If such a crossing could be located, NRD by this route would have to compete with internal conversion to the ^1^nπ* state, but we would expect this spin-allowed transition to occur more quickly than the spin-forbidden ISC to a ^3^nπ* state.

A total of three CIs between the two lowest energy excited singlet states were found, two directly by searching for one starting at the FC geometries (*anti*-1^1^ππ*/2^1^ππ* and *syn*-1^1^ππ*/2^1^nπ*) and the third in the search for crossing using UKS TDDFT (*anti*-1^1^nπ*/2^1^ππ*). Considering first the *anti*-1^1^ππ*/2^1^ππ* point, this has an energy directly between the energies of the 1^1^ππ* and 2^1^ππ* states at the *anti*-FC geometry, corresponding to 2800 cm^−1^ above the 1^1^ππ* excitation point. The crossing would thus likely be energetically accessible in the experiment, however, we see that the character of the two states is ^1^ππ*, i.e., it does not allow for crossing to the ^1^nπ* state. We also note that the final geometry has a *syn* conformation; the OH bond now points in the opposite direction to its original position. Next, we consider the CIs labelled *syn*-1^1^ππ*/2^1^nπ* and *anti*-1^1^nπ*/2^1^ππ*, both of which have very similar energies (separated by about 50 cm^−1^) suggesting that the main difference between them is the orientation of the OH bond. The crossings are below the energies of the 1^1^ππ* state at either FC point, so are definitely energetically accessible after excitation. With regard to the state characters, both crossings are between the ^1^nπ* state (greater than 85% components in both cases) and the bright ^1^ππ* state, so they represent an accessible route from the initially excited state and the ^1^nπ* state which can, later on, give access to the triplet manifold.

We have thus found a possible path from the bright 1^1^ππ* state to the ^3^ππ* manifold and we also note that there are decreasing energy CIs between the triplet states which provide a path from T_5_ to T_2_, so there is a path towards the ground state. We have been unable to locate a crossing between the lowest two energy triplet states (except a very high energy crossing at a distorted geometry with the alkene-ester chain having migrated around the ring); however, that is not to say that one does not exist. With regard to the characters of the states at these crossing points: only the *anti*-3^1^nπ*/4^3^nπ* crossing involves the ^3^nπ* state. For the *syn* conformations, all states are ^3^ππ* at the triplet CIs, apart from the fourth triplet at the *syn*-4^3^nπ*/5^3^ππ* CI. As the crossing from the singlet manifold is ^1^nπ* to ^3^ππ*, it is not necessary for the decaying molecule to cross to the ^3^nπ* at these CIs, the nuclear wavepacket may stay on the ^3^ππ* state as it makes its way down towards the lower states. Transfer of density to the ^3^nπ* state may compete with the decay through the ^3^ππ* states, slowing the NRD.

As a final set of calculations, we searched for barriers between relevant geometries. Of first interest was any barrier between the CIs found between the two lowest energy singlets (*syn*-1^1^ππ*/2^1^nπ* and *anti*-1^1^nπ*/2^1^ππ*) and their corresponding FC geometries on the first singlet excited state potential energy surface. In both cases, the energies found were very close to that of the 1^1^ππ* minimum (∼2500 cm^−1^ below the 1^1^ππ* energy at the FC point), suggesting that no barrier is present. We note that there is also no barrier to the 1^1^ππ* minimum energy geometry, thus there is likely to be competition between relaxation for the 1^1^ππ* minimum and to the singlet CIs, potentially slowing the NRD. A barrier was also sought between the *syn*-1^1^ππ*/2^1^nπ* geometry and the *syn*-1^1^nπ*/5^3^ππ* point (i.e., the location of the largest SOC) and, again, none was found. This indicates that the path from the FC excitation geometry to the point of most likely ISC (1^1^nπ* to 5^3^ππ*) is barrierless, the rate of NRD being determined by coupling between the states and the rate of competitive processes including relaxation to the 1^1^ππ* minimum.

**Figure 8:**
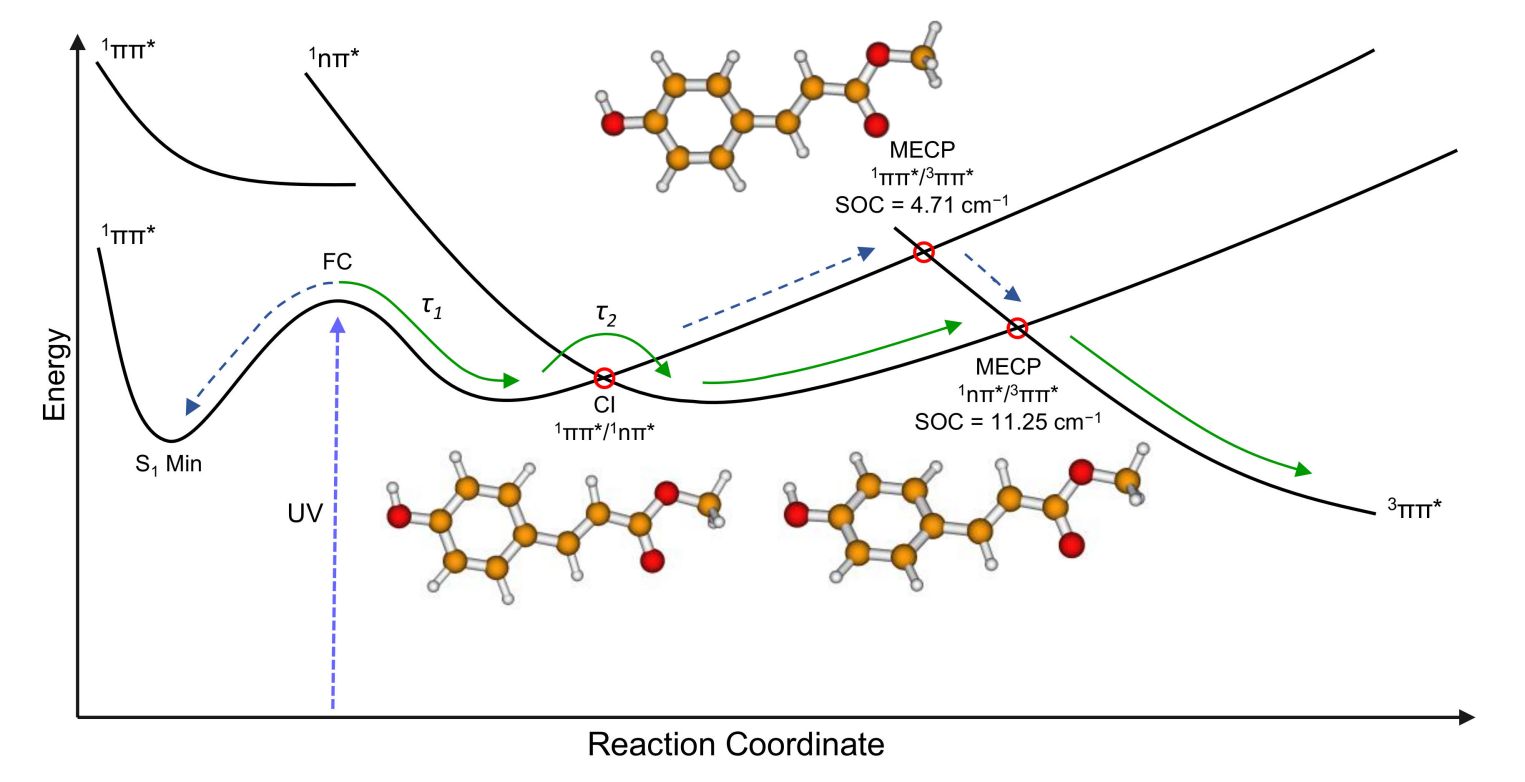
Potential energy surface schematic for s-cis OH-syn p-HMC, based on the energy landscape diagram in Figure 6. At the point of excitation, the vertical ordering of states is the first 1ππ* state, the second 1ππ* (not of dynamical interest) and the 1nπ* state. The green arrows represent the major NRD route after photoexcitation to the first 1ππ* state, while the dark blue dotted arrows represent alternative or competing excited state processes. The processes identified experimentally are labelled τ1 and τ2 and relate to the lifetimes measured in Section 2.1. CI and MECP structures are shown along with the relevant spin-orbit coupling (SOC) values.

Bringing together the experimental and computational work, Figure 8 schematically illustrates the calculated major NRD route for s-*cis* OH-*syn p*-HMC along with the associated lifetimes, *τ*_1_ and *τ*_2_, determined by TR-IY and TR-PES. We focus specifically on the *syn* conformer since it shows a clearer path, with strong SOC, down through the inter-state crossings. Upon photoexcitation using a 308.5 nm pump, we propose that s-*cis p*-HMC evolves out of the FC region with no energy barrier in ∼150 fs. Subsequent to this, internal conversion to the ^1^nπ* state occurs within ∼2.5 ps, and finally ISC to the ^3^ππ* state with no energy barrier.

## 3. Materials and Methods

### 3.1. Experimental Setup

The experimental setup has been described in detail elsewhere [23,33]. To briefly summarise, a Ti:Sapphire oscillator (Spectra-Physics Tsunami) and regenerative amplifier (Spectra-Physics Spitfire XP) are used to produce ∼40 fs laser pulses at a rate of 1 kHz and centred around 800 nm. The output laser beam is ∼3 W and is subsequentially split into three equal ∼1 W parts. One 1 W part pumps an optical parametric amplifier (Light Conversion TOPAS-C) to produce the pump pulse, centred at 308.5 nm to ensure s-*cis* conformer selective excitation of *p*-HMC to the S_1_(ππ*) state. Another 1 W part either pumps a second TOPAS-C to generate the 240 nm probe or is successively frequency converted using a series of type I, type II and type I β-barium borate crystals to produce the 200 nm probe. For simplicity, the polarisations of the pump and probe are parallel with respect to each other, and the dynamics appear unaffected upon changing the probe to magic angle (54.7°) with respect to the pump—indicating the absence of rotational artifacts (ESI, Figure S3).

Both beams intersect a molecular beam produced by seeding *p*-HMC (99.9% purity by HPLC, Apollo Scientific) heated to 170 °C into 1.5, 3 and 5 bar helium. This gas mixture is expanded into vacuum via an Even-Lavie pulsed solenoid valve and is subsequentially passed through a 2 mm diameter skimmer [34,35].

For TR-IY, the photoions are accelerated, via an electric field, towards a detector which consists of a 25 mm active area MCP with a metal anode detector (Del Mar Photonics MCP-MA25/2). The signal output is measured by a digital oscilloscope (LeCroy LT372 Waverunner) and the ion-signal relating to the parent ion (*p*-HMC^+^) is monitored as a function of Δ*t*; this creates the TR-IY transients.

A velocity map ion imaging setup, based off a design by Eppink and Parker, is used for TR-PES [36]. The photoelectrons are accelerated and focused onto a position-sensitive temporally-gated detector, consisting of two MCPs coupled to a phosphor screen (Photek VID-240) and imaged by a CCD camera (Basler A-312f). In this configuration, electrons with the same initial velocity are mapped onto the same point on the detector. The two-dimensional images generated are used to reconstruct the original three-dimensional Newton sphere using a polar onion peeling algorithm, from which the desired one-dimensional photoelectron spectrum is derived [37]. Xenon is used to calibrate the detector by which the obtained photoelectron spectrum is scaled to coincide with the energy of its well-known ionisation states [38].

Dynamical information from the TR-IY transients (*S*(Δ*t*) vs. Δ*t*) was extracted via a non-linear curve fitting algorithm consisting of a sum of exponential decay functions convoluted with the instrument response function (IRF):

(2) $$S\left(\Delta t\right)=S_{0}+G\left(\Delta t\right)*\left[\underset{i}{\sum }A_{i}e^{\left(-\frac{\Delta t-t_{0}\;}{\tau_{i}}\right)}H(\Delta t-t_{0})\right]\;$$

Here, *S*_0_ is the signal baseline, *G*(Δ*t*) is the Gaussian function to account for the IRF, *A_i_* is the amplitude of the *i*-decay, *t*_0_ is time zero, *τ_i_* is the lifetime of the *i*-decay and *H*(Δ*t* − *t*_0_) is a step function that is used to define time zero as the starting point of all observed dynamics; such that:

(3) $$H\left(\Delta t-t_{0}\right)=\left\{\begin{array}{c} 0\;\;\;if\;\left(\Delta t-t_{0}\right)<0,\\ 1\;\;\;if\;\left(\Delta t-t_{0}\right)\geq 0. \end{array}\right.$$

The instrument response (IR) is estimated from the cross-correlation of the 308.5 nm pump and 240/200 nm probe with ammonia (ESI, Figures S4 and S5). From this, the IR is determined to be 117 ± 3 fs and 154 ± 6 fs with the 240 nm and 200 nm probe, respectively.

### 3.2. Computational Details

To further investigate the NRD of *p*-HMC, we have performed a series of electronic structure calculations to explore the ground- and excited-state potential energy surfaces of the molecule. To do so, we have used DFT for the ground state and TDDFT for the excited states, as implemented in the ORCA quantum chemistry package (version 5.0.1) [39,40,41], specifically using the ωB97X functional, both in RKS and UKS forms allied with the 6-311G(d,p) basis set [42,43]. The molecular geometry has been optimised using RKS DFT and TDDFT in order to locate energy minima on the electronic ground and excited states (both singlet and triplet); at such points, frequency calculations were carried out to ensure that the geometries were indeed minima of the potential energy surfaces, rather than saddle points. Additionally, MECPs between excited states were located, these being CIs in the case of crossing between states of the same multiplicity (found using RKS TDDFT). Once these minima and MECPs had been found, further calculations were performed to locate TSs between selected geometries, on the states of interest, where such barriers exist. The initial guess for the TS structure was found by taking the central geometry in a linear interpolation between the two selected geometries (using their respective Z-matrices). Upon convergence of the TS structures using the energies of the state of interest, frequency calculations were performed on that state to ensure that all frequencies were real except for one; the single imaginary frequency indicates that the structure is indeed a TS. All such optimised geometries were indeed found to be TSs using this condition. At points of crossing between singlet and triplet states, SOCs were calculated to assess the probability of ISC at such geometries. These points were located using UKS TDDFT, but were then checked using RKS TDDFT, where state energies were found to be the same as when using UKS; the SOCs of these RKS states found with quasi-degenerate perturbation theory [44]. Tight convergence criteria were used both for the geometry optimisations (convergence criteria were: 10^−6^ Eh change in energy; 10^−4^ Eh/Bohr maximum absolute gradient component; 3 × 10^−5^ Eh/Bohr root mean square (RMS) of gradient components; 10^−3^ Bohr maximum coordinate displacement and 6 × 10^−4^ Bohr RMS displacement) and the underlying electronic structure calculations (convergence criteria were: 10^−8^ Eh energy change; 10^−5^ Eh change in the one-electron component of the energy; 10^−5^ orbital gradient, 10^−5^ orbital rotation angle; and 5 × 10^−7^ error in the direct inversion of the iterative subspace).

## 4. Conclusions

We have investigated the gas-phase excited state dynamics of s-*cis para*-hydroxy methylcinnamate with both experiment and computation. Using pump-probe femtosecond time-resolved ion-yield and time-resolved photoelectron spectroscopies, the gas-phase S_1_(ππ*) excited state lifetime was determined. Time-resolved ion-yield transients produced S_1_(ππ*) lifetimes of 2.15 ± 0.18 ps and 2.46 ± 0.35 ps when probing at 240 nm and 200 nm, respectively. These lifetimes sensibly compare with time-resolved photoelectron spectroscopy measurements. Furthermore, the possible relaxation pathways were calculated by time-dependent density functional theory. We found the major non-radiative route, following photoexcitation to the S_1_(ππ*) state, is mostly likely intersystem crossing to the ^3^ππ* state, mediated by an optically dark ^1^nπ* state. Overall, the excited state lifetime and major non-radiative decay route from the first excited state in s-*cis para*-hydroxy methylcinnamate correlates strongly with structurally similar molecules, such as *para*-coumaric acid and methylcinnamate. This conclusion brings us closer to developing a greater understanding for the photodynamical behaviour of cinnamates and coumaric acids, to ultimately tailor similar molecules for applications including sunscreens and molecular heaters.

## Figures and Tables

**Table 1:** Spin-orbit couplings between singlet and triplet states of *p*-HMC calculated at MECPs found by optimising geometries from both the *anti* and *syn* conformer FC points. Matrix elements for the x, y and z components (all imaginary) given along with the magnitudes.

Initial Geometry	MECP	Spin Orbit Coupling Values (cm^−1^)			
		S→T (x)	S→T (y)	S→T (z)	Magnitude
*Anti*	1^1^ππ*/3^3^ππ* (S_1_/T_3_)	0.66i	0.52i	−0.95i	1.27
	1^1^ππ*/6^3^ππ* (S_1_/T_6_)	0.01i	0.00	−0.02i	0.02
	2^1^ππ*_2_/6^3^ππ* (S_2_/T_6_)	0.00	0.00	0.01i	0.01
	3^1^ππ*/7^3^ππ* (S_3_/T_7_)	0.00	0.00	−0.01i	0.01
*Syn*	1^1^ππ*/3^3^ππ* (S_1_/T_3_)	0.02i	0.71i	−0.06i	0.71
	1^1^ππ*/4^3^ππ* (S_1_/T_4_)	0.19i	−0.53i	−0.02i	0.56
	1^1^nπ*/5^3^ππ* (S_1_/T_5_)	4.29i	10.28i	1.58i	11.25
	2^1^ππ*/5^3^ππ* (S_2_/T_5_)	−0.59i	4.57i	0.99i	4.71
	2^1^ππ*/6^3^ππ* (S_2_/T_6_)	0.01i	0.00	0.00	0.01
	3^1^ππ*/7^3^ππ* (S_3_/T_7_)	−0.01i	0.00	0.00	0.01

## Data Availability

The data presented in this study is available in the article or supplementary material.

## Supplementary Materials

The following are available online. Figures S1 and S2: TR-IY transients up to 900 ps, Figure S3: TR-IY transient at magic angle, Figures S4 and S5: cross-correlation, Figure S6: molecular orbitals, Tables S1–S5: computational data.

## References

[1] Kinoshita S.N., Harabuchi Y., Inokuchi Y., Maeda S., Ehara M., Yamazaki K., Ebata T. (2021). Substitution Effect on the Nonradiative Decay and Trans Cis Photoisomerization Route: A Guideline to Develop Efficient Cinnamate-Based Sunscreens. Phys. Chem. Chem. Phys..

[2] Promkatkaew M., Suramitr S., Karpkird T., Wanichwecharungruang S., Ehara M., Hannongbua S. (2014). Photophysical Properties and Photochemistry of Substituted Cinnamates and Cinnamic Acids for UVB Blocking: Effect of Hydroxy, Nitro, and Fluoro Substitutions at Ortho, Meta, and Para Positions. Photochem. Photobiol. Sci..

[3] Peperstraete Y., Staniforth M., Baker L.A., Rodrigues N.D.N., Cole-Filipiak N.C., Quan W.D., Stavros V.G. (2016). Bottom-up Excited State Dynamics of Two Cinnamate-Based Sunscreen Filter Molecules. Phys. Chem. Chem. Phys..

[4] Karpkird T.M., Wanichweacharungruang S., Albinsson B. (2009). Photophysical Characterization of Cinnamates. Photochem. Photobiol. Sci..

[5] Iida Y., Kinoshita S.N., Kenjo S., Muramatsu S., Inokuchi Y., Zhu C., Ebata T. (2020). Electronic States and Nonradiative Decay of Cold Gas-Phase Cinnamic Acid Derivatives Studied by Laser Spectroscopy with a Laser-Ablation Technique. J. Phys. Chem. A.

[6] Meyer T.E. (1985). Isolation and Characterization of Soluble Cytochromes, Ferredoxins and Other Chromophoric Proteins from the Halophilic Phototrophic Bacterium Ectothiorhodospira Halophila. Biochim. Biophys. Acta.

[7] Meyer T.E., Fitch J.C., Bartsch R.G., Tollin G., Cusanovich M.A. (1990). Soluble Cytochromes and a Photoactive Yellow Protein Isolated from the Moderately Halophilic Purple Phototrophic Bacterium, Rhodospirillum Salexigens. Biochim. Biophys. Acta.

[8] Meyer T.E., Yakali E., Cusanovich M.A., Tollin G. (1987). Properties of a Water-Soluble, Yellow Protein Isolated from a Halophilic Phototrophic Bacterium That Has Photochemical Activity Analogous to Sensory Rhodopsin. Biochemistry.

[9] Imamoto Y., Kataoka M. (2007). Structure and Photoreaction of Photoactive Yellow Protein, a Structural Prototype of the PAS Domain Superfamily. Photochem. Photobiol..

[10] Carroll E.C., Hospes M., Valladares C., Hellingwerf K.J., Larsen D.S. (2011). Is the Photoactive Yellow Protein a UV-B/Blue Light Photoreceptor?. Photochem. Photobiol. Sci..

[11] Schlichting I., Berendzen J. (1997). Out of the Blue: The Photocycle of the Photoactive Yellow Protein. Structure.

[12] Sprenger W.W., Hoff W.D., Armitage J.P., Hellingwerf K.J. (1993). The Eubacterium Ectothiorhodospira Halophila Is Negatively Phototactic, with a Wavelength Dependence That Fits the Absorption Spectrum of the Photoactive Yellow Protein. J. Bacteriol..

[13] Burnett M.E., Hu J.Y., Wang S.Q. (2012). Sunscreens: Obtaining Adequate Photoprotection. Dermatol. Ther..

[14] Mancuso J.B., Maruthi R., Wang S.Q., Lim H.W. (2017). Sunscreens: An Update. Am. J. Clin. Dermatol..

[15] Palm M.D., O’Donoghue M.N. (2007). Update on Photoprotection. Dermatol. Ther..

[16] De Groot M., Gromov E.V., Köppel H., Buma W.J. (2008). High-Resolution Spectroscopy of Methyl 4-Hydroxycinnamate and Its Hydrogen-Bonded Water Complex. J. Phys. Chem. B.

[17] Roberts G.M., Stavros V.G. (2014). The Role of Πσ* States in the Photochemistry of Heteroaromatic Biomolecules and Their Subunits: Insights from Gas-Phase Femtosecond Spectroscopy. Chem. Sci..

[18] Sobolewski A.L., Domcke W., Dedonder-Lardeux C., Jouvet C. (2002). Excited-State Hydrogen Detachment and Hydrogen Transfer Driven by Repulsive 1πσ* States: A New Paradigm for Nonradiative Decay in Aromatic Biomolecules. Phys. Chem. Chem. Phys..

[19] Ratzer C., Küpper J., Spangenberg D., Schmitt M. (2002). The Structure of Phenol in the S1-State Determined by High Resolution UV-Spectroscopy. Chem. Phys..

[20] Dixon R.N., Oliver T.A.A., Ashfold M.N.R. (2011). Tunnelling under a Conical Intersection: Application to the Product Vibrational State Distributions in the UV Photodissociation of Phenols. J. Chem. Phys..

[21] Smolarek S., Vdovin A., Tan E.M.M., De Groot M., Buma W.J. (2011). Spectroscopy and Dynamics of Methyl-4-Hydroxycinnamate: The Influence of Isotopic Substitution and Water Complexation. Phys. Chem. Chem. Phys..

[22] Smolarek S., Vdovin A., Perrier D.L., Smit J.P., Drabbels M., Buma W.J. (2010). High-Resolution Excitation and Absorption Spectroscopy of Gas-Phase p-Coumaric Acid: Unveiling an Elusive Chromophore. J. Am. Chem. Soc..

[23] Krokidi K.M., Turner M.A.P., Pearcy P.A.J., Stavros V.G. (2021). A Systematic Approach to Methyl Cinnamate Photodynamics. Mol. Phys..

[24] Kinoshita S.N., Inokuchi Y., Onitsuka Y., Kohguchi H., Akai N., Shiraogawa T., Ehara M., Yamazaki K., Harabuchi Y., Maeda S. (2019). The Direct Observation of the Doorway 1nπ∗ State of Methylcinnamate and Hydrogen-Bonding Effects on the Photochemistry of Cinnamate-Based Sunscreens. Phys. Chem. Chem. Phys..

[25] Yamazaki K., Miyazaki Y., Harabuchi Y., Taketsugu T., Maeda S., Inokuchi Y., Kinoshita S.N., Sumida M., Onitsuka Y., Kohguchi H. (2016). Multistep Intersystem Crossing Pathways in Cinnamate-Based UV-B Sunscreens. J. Phys. Chem. Lett..

[26] Tan E.M.M., Amirjalayer S., Bakker B.H., Buma W.J. (2013). Excited State Dynamics of Photoactive Yellow Protein Chromophores Elucidated by High-Resolution Spectroscopy and Ab Initio Calculations. Faraday Discuss..

[27] Shimada D., Kusaka R., Inokuchi Y., Ehara M., Ebata T. (2012). Nonradiative Decay Dynamics of Methyl-4-Hydroxycinnamate and Its Hydrated Complex Revealed by Picosecond Pump-Probe Spectroscopy. Phys. Chem. Chem. Phys..

[28] Kinoshita S.N., Miyazaki Y., Sumida M., Onitsuka Y., Kohguchi H., Inokuchi Y., Akai N., Shiraogawa T., Ehara M., Yamazaki K. (2018). Different Photoisomerization Routes Found in the Structural Isomers of Hydroxy Methylcinnamate. Phys. Chem. Chem. Phys..

[29] Kotsina N., Candelaresi M., Saalbach L., Zawadzki M.M., Crane S.W., Sparling C., Townsend D. (2020). Short-Wavelength Probes in Time-Resolved Photoelectron Spectroscopy: An Extended View of the Excited State Dynamics in Acetylacetone. Phys. Chem. Chem. Phys..

[30] De Camillis S., Miles J., Alexander G., Ghafur O., Williams I.D., Townsend D., Greenwood J.B. (2015). Ultrafast Non-Radiative Decay of Gas-Phase Nucleosides. Phys. Chem. Chem. Phys..

[31] Liu Y.-Z., Qin C.-C., Zhang S., Wang Y.-M., Zhang B. (2011). Ultrafast Dynamics of the First Excited State of Chlorobenzene. Acta Phys. Chim. Sin..

[32] El-Sayed M.A. (1963). Spin—Orbit Coupling and the Radiationless Processes in Nitrogen Heterocyclics. J. Chem. Phys..

[33] Iqbal A., Pegg L.J., Stavros V.G. (2008). Direct versus Indirect H Atom Elimination from Photoexcited Phenol Molecules. J. Phys. Chem. A.

[34] Even U. (2015). The Even-Lavie Valve as a Source for High Intensity Supersonic Beam. EPJ Tech. Instrum..

[35] Even U., Jortner J., Noy D., Lavie N., Cossart-Magos C. (2000). Cooling of Large Molecules below 1 K and He Clusters Formation. J. Chem. Phys..

[36] Eppink A.T.J.B., Parker D.H. (1997). Velocity Map Imaging of Ions and Electrons Using Electrostatic Lenses: Application in Photoelectron and Photofragment Ion Imaging of Molecular Oxygen. Rev. Sci. Instrum..

[37] Roberts G.M., Nixon J.L., Lecointre J., Wrede E., Verlet J.R.R. (2009). Toward Real-Time Charged-Particle Image Reconstruction Using Polar Onion-Peeling. Rev. Sci. Instrum..

[38] Compton R.N., Miller J.C., Carter A.E., Kruit P. (1980). Resonantly Enhanced Multiphoton Ionization of Xenon: Photoelectron Energy Analysis. Chem. Phys. Lett..

[39] Neese F. (2012). The ORCA Program System. Wiley Interdiscip. Rev. Comput. Mol. Sci..

[40] Ekström U., Visscher L., Bast R., Thorvaldsen A.J., Ruud K. (2010). Arbitrary-Order Density Functional Response Theory from Automatic Differentiation. J. Chem. Theory Comput..

[41] Weigend F. (2006). Accurate Coulomb-Fitting Basis Sets for H to Rn. Phys. Chem. Chem. Phys..

[42] Krishnan R., Binkley J.S., Seeger R., Pople J.A. (1980). Self-Consistent Molecular Orbital Methods. XX. A Basis Set for Correlated Wave Functions. J. Chem. Phys..

[43] Frisch M.J., Pople J.A., Binkley J.S. (1984). Self-Consistent Molecular Orbital Methods 25. Supplementary Functions for Gaussian Basis Sets. J. Chem. Phys..

[44] De Souza B., Farias G., Neese F., Izsák R. (2019). Predicting Phosphorescence Rates of Light Organic Molecules Using Time-Dependent Density Functional Theory and the Path Integral Approach to Dynamics. J. Chem. Theory Comput..

