# Supplementary material for: Experimental and Computational Analysis of Para-Hydroxy Methylcinnamate following Photoexcitation
Source: Molecules. 2021 Dec 15;26(24):7621. doi: 10.3390/molecules26247621 (PMC8704431; doi:10.3390/molecules26247621)
Supplement: Supplementary file 1 [file molecules-26-07621-s001.zip › molecules-1505253-supplementary.pdf]

Electronic Supplementary Information for:

Experimental and Computational Analysis of *Para*-Hydroxy Methylcinnamate Following Photoexcitation

Jack Dalton,[1] Gareth W. Richings,[1] Jack M. Woolley,[1] Temitope T. Abiola,[1] Scott Habershon[1,*] and Vasilios G. Stavros[1,*]

[1] Department of Chemistry, University of Warwick, Gibbet Hill Road, Coventry, CV4 7AL, UK.

***** Correspondence: S.Habershon@warwick.ac.uk and V.Stavros@warwick.ac.uk

Section 1: Experimental

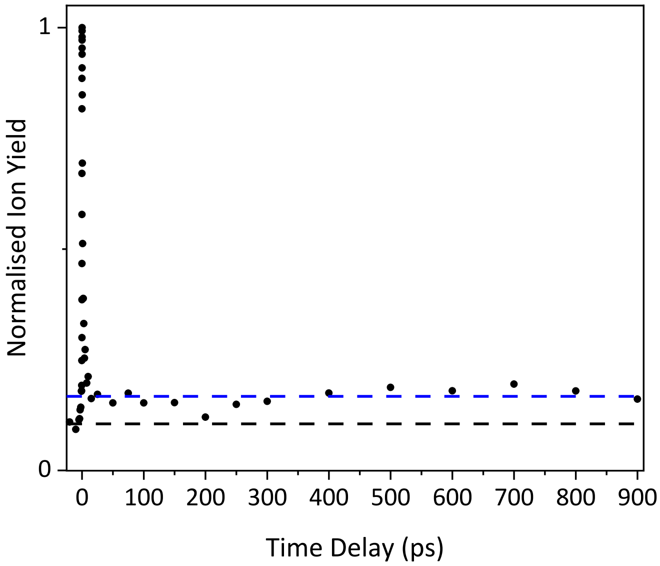

Figure S1. TR-IY transient of *p*-HMC at 308.5 nm pump and 240 nm probe to show the long-lived feature extending beyond 900 ps. The black dashed line shows the signal baseline, and the blue dashed line shows the baseline offset of the long-lived feature.

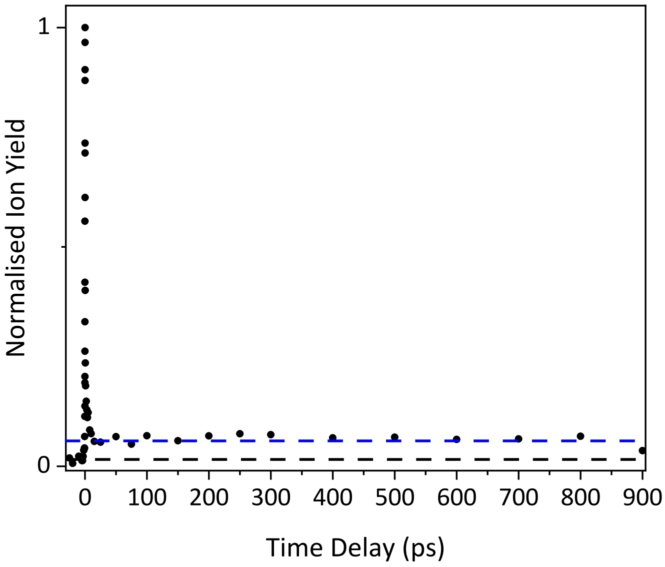

Figure S2. TR-IY transient of *p*-HMC at 308.5 nm pump and 200 nm probe to show the long-lived feature extending beyond 900 ps. The black dashed line shows the signal baseline, and the blue dashed line shows the baseline offset of the long-lived feature.

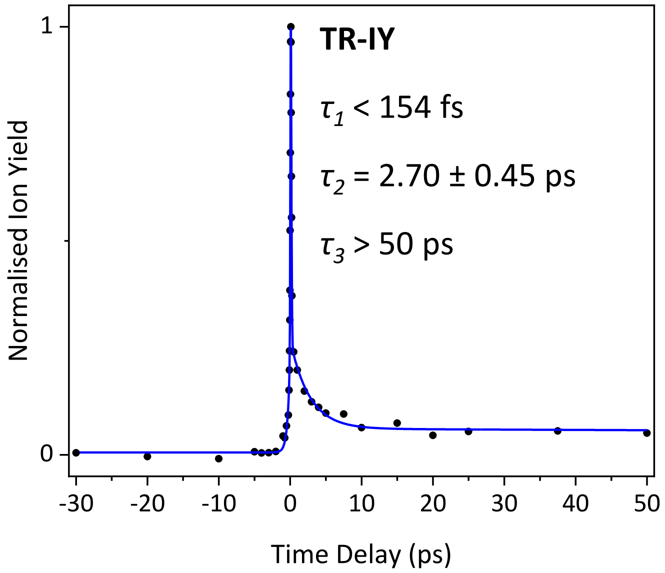

Figure S3. TR-IY transient of *p*-HMC at 308.5 nm pump and 200 nm probe with a 1.5 bar helium backing pressure. The polarisations of the pump and probe are at magic angle (54.7^{o}) with respect to each other to avoid rotational artifacts resulting from laser induced transition dipole moment alignment. The lifetimes are within error of the lifetimes produced from parallel pump-probe polarisations (Figure 3 of manuscript).

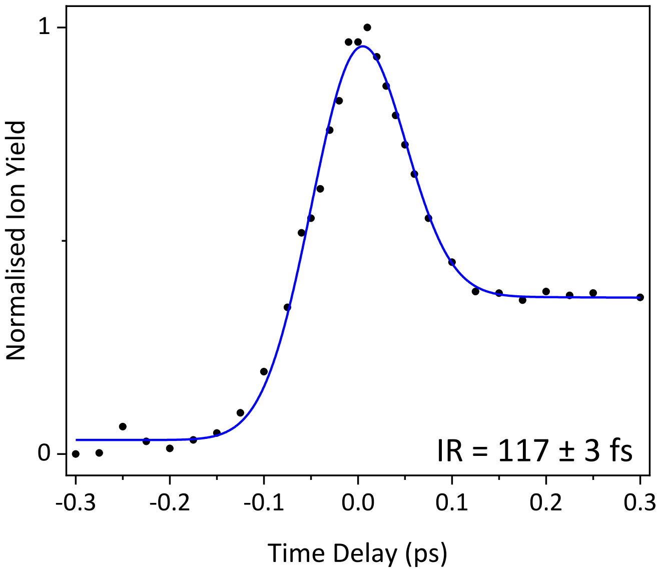

Figure S4. 308.5 nm pump and 240 nm probe TR-IY cross-correlation with ammonia to estimate the temporal resolution of the experiment. Here, 5% ammonia/helium gas mixture is expanded into vacuum via the Even-Lavie pulsed solenoid valve and subsequentially excited and ionised with varying pump-probe time delays. The baseline offset is likely due to population of a long-lived Rydberg state with the pump[1]. The data is fitted with a Gaussian and a single (positive going) decay lifetime of 30 ps to account for the long-lived state population. This has been achieved using equation (2) from the manuscript plus a Gaussian function. The full width half maximum (FWHM) produced from the Gaussian corresponds to the IR (117 fs).

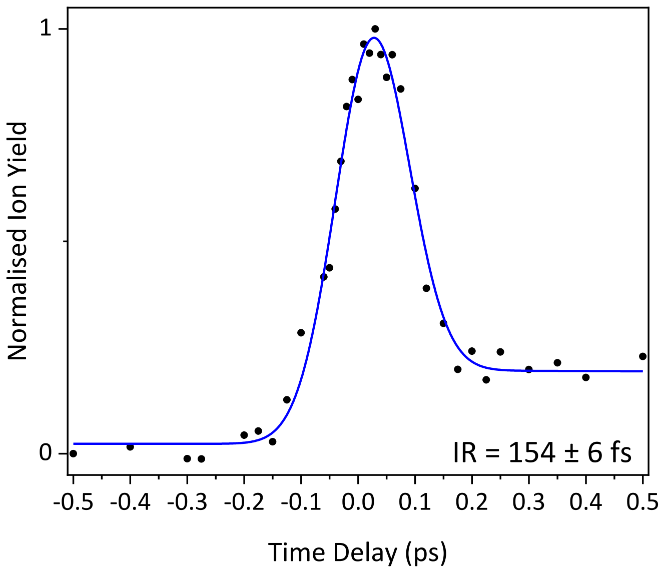

Figure S5. 308.5 nm pump and 200 nm probe TR-IY cross-correlation with ammonia to estimate the temporal resolution of the experiment. The method is analogous to the cross-correlation with 240 nm. The baseline offset is still seen with 200 nm, further indicating population of a long-lived Rydberg state with the pump. The extracted IR is 154 fs.

Section 2: Computational

Table S1. Energies of the ground and excited states (first three singlet and first seven triplet) of *p*-HMC optimised from the s-*cis* OH-*anti* conformer using ωB97X (TD)DFT/6-311G(d,p) as implemented in ORCA 5.0.1[2–6]. Energies are relative to the ground state at the Franck-Condon point (S_0 minimum). NA indicates that the labelled minimum was sought but not located. The minima and conical intersections were located using restricted Kohn-Sham TDDFT whilst those state crossings labelled X_i/X_j (including those which are actually conical intersections) were found using unrestricted Kohn-Sham TDDFT.

	Relative Energies (cm^{-1})										
**Optimised Geometry**	**S_0**	**S_1**	**S_2**	**S_3**	**T_1**	**T_2**	**T_3**	**T_4**	**T_5**	**T_6**	**T_7**
S_0 Minimum	0	39993	41952	42588	24783	35026	35887	38613	39471	41484	46640
S_1 Minimum	2517	37512	42121	42529	21167	35892	36696	39295	40360	42933	46681
S_2 Minimum	1108	39058	40838	42899	23422	34043	35585	38088	39892	40428	47109
S_3 Minimum	NA										
T_1 Minimum	4294	38165	42968	43993	20545	37204	38527	40289	41853	44084	48101
T_2 Minimum	2942	41436	43539	45174	26850	32253	38257	39717	42077	43551	49381
T_3 Minimum	1199	39048	41461	42290	23541	34669	35282	37462	39307	40880	46478
T_4 Minimum	NA										
T_5 Minimum	NA										
T_6 Minimum	NA										
T_7 Minimum	2860	39870	40619	43747	24855	36939	36994	37932	40293	42144	42365
S_1/S_2 Conical Intersection	2041	41300	41307	44590	26290	34062	35668	38705	41404	42135	48649
S_2/S_3 Conical Intersection	14841	47335	48557	48577	26178	44824	45899	48409	50343	53285	53968
T_1/T_2 Conical Intersection	NA										
T_2/T_3 Conical Intersection	1756	40561	42482	44155	26279	33985	33999	39364	41035	41617	48286
T_3/T_4 Conical Intersection	1789	38990	39486	42472	23994	35670	35954	35967	39318	41817	43692
T_4/T_5 Conical Intersection	3045	40512	41916	43691	24597	36455	36753	39513	39519	40742	47839
T_5/T_6 Conical Intersection	20019	50704	61958	64427	36833	47238	56705	62207	62697	62699	70002
T_6/T_7 Conical Intersection	2897	39885	40472	43900	24853	36879	37055	38099	40482	42303	42308
X_1/X_2 Crossing (T_1/T_2)	NA										
X_2/X_3 Crossing (T_2/T_3)	1836	40591	42546	44254	26337	33982	34001	39451	41145	41667	48392
X_3/X_4 Crossing (S_1/T_3)	3280	37990	42435	43462	21875	35113	37993	39567	41424	43763	47010
X_4/X_5 Crossing (T_4/T_5)	1205	39052	40637	41599	23476	35052	35307	37645	37648	40837	44918
X_5/X_6 Crossing (S_1/S_2)	2851	38662	38664	43406	22416	35817	36286	37655	40628	42670	43669
X_6/X_7 Crossing (S_1/T_6)	1794	39464	41473	42347	23271	35781	36046	38626	39424	39474	46517
X_7/X_8 Crossing (S_2/T_6)	1166	39076	40873	43153	23531	33842	35736	38242	40131	40882	47380
X_8/X_9 Crossing (S_2/S_3)	1025	38837	41044	41053	23170	34481	35419	38006	38060	40377	45340
X_9/X_{10} Crossing (S_3/T_7)	1968	39194	39734	42691	24106	36201	36202	36504	39260	41634	42684

Table S2. Energies of the ground and excited states (first three singlet and first seven triplet) of *p*-HMC optimised from the s-*cis* OH-*syn* conformer using ωB97X (TD)DFT/6-311G(d,p) as implemented in ORCA 5.0.1[2–6]. Energies are relative to the ground state at the Franck-Condon point (S_0 minimum) for the s-*cis* OH-*anti* conformer (Table S1). NA indicates that the labelled minimum was sought but not located. The minima and conical intersections were located using restricted Kohn-Sham TDDFT whilst those state crossings labelled X_i/X_j (including those which are actually conical intersections) were found using unrestricted Kohn-Sham TDDFT.

	Relative Energies/cm^{-1}										
**Optimised Geometry**	**S_0**	**S_1**	**S_2**	**S_3**	**T_1**	**T_2**	**T_3**	**T_4**	**T_5**	**T_6**	**T_7**
S_0 Minimum	38	39882	41984	42620	24725	35077	35831	38831	39514	41366	46668
S_1 Minimum	2594	37378	42199	42635	21111	35709	36901	39384	40786	42815	46781
S_2 Minimum	1196	39049	40867	42995	23522	34050	35547	38286	39975	40473	47187
S_3 Minimum	NA										
T_1 Minimum	4081	37966	42891	43883	20487	36904	38427	40213	41956	43732	47990
T_2 Minimum	2708	41038	42686	44176	25544	32573	37978	39840	41128	43061	48430
T_3 Minimum	NA										
T_4 Minimum	1013	39756	41416	42174	24640	34378	34694	37853	39134	40808	46285
T_5 Minimum	NA										
T_6 Minimum	NA										
T_7 Minimum	NA										
S_1/S_2 Conical Intersection	2943	38618	38629	43522	22438	35775	36185	37860	41044	42520	43656
S_2/S_3 Conical Intersection	1070	38780	41052	41067	23163	34455	35397	38071	38238	40291	45340
T_1/T_2 Conical Intersection	43764	70530	74495	75738	54902	54958	65547	73365	75531	76888	81144
T_2/T_3 Conical Intersection	1831	40618	42565	44264	26372	34007	34013	39577	41160	41468	48381
T_3/T_4 Conical Intersection	3967	40609	43547	44348	25254	36111	38823	38827	41374	42158	48458
T_4/T_5 Conical Intersection	979	39090	40941	41442	23651	34611	35075	37939	37951	40521	45176
T_5/T_6 Conical Intersection	1851	39602	41563	42230	23626	35591	36064	38858	39283	39302	46378
T_6/T_7 Conical Intersection	2891	39859	40517	43879	24882	36837	37040	38052	40464	42269	42312
X_1/X_2 Crossing (T_1/T_2)	NA										
X_2/X_3 Crossing (T_2/T_3)	1912	40660	42642	44360	26464	34004	34013	39664	41256	41532	48483
X_3/X_4 Crossing (S_1/T_3)	2939	37480	42522	42913	21404	35676	37490	39752	40852	43320	47187
X_4/X_5 Crossing (S_1/T_4)	3390	37931	40612	44185	24671	35181	36765	37930	40951	43094	43407
X_5/X_6 Crossing (S_1/T_5)	2133	39053	41264	42997	25765	35533	36248	36385	39050	41996	43745
X_6/X_7 Crossing (S_2/T_5)	2982	38407	40162	43605	23910	35764	36571	37804	40160	42308	43419
X_7/X_8 Crossing (S_2/T_6)	1251	39124	40878	43251	23634	33863	35697	38418	40235	40881	47468
X_8/X_9 Crossing (T_6/T_7)	2998	39864	40422	43929	24784	36924	36987	38193	40517	42283	42286
X_9/X_{10} Crossing (S_3/T_7)	2013	39216	39640	42720	24076	35975	36227	36702	39497	41517	42714

Table S3. Energies of the ground and excited states (first three singlet and first seven triplet) of *p*-HMC optimised during transition state searches between geometries found between s-*cis* OH-*anti* conformers (Table S1) using ωB97X (TD)DFT/6-311G(d,p) as implemented in ORCA 5.0.1[2–6]. Energies are relative to the ground state at the Franck-Condon point (S_0 minimum) for the s-*cis* OH-*anti* conformer (Table S1).

	Relative Energies (cm^{-1})										
**Optimised Geometry**	S_0	S_1	S_2	S_3	T_1	T_2	T_3	T_4	T_5	T_6	T_7
S_0 Min. → S_1 Min. TS (S_1)	2522	37502	42125	42526	21162	35886	36693	39301	40362	42925	46676
S_0 Min. → S_2 Min. TS (S_2)	1115	39059	40838	42906	23428	34039	35575	38087	39902	40434	47100
S_0 Min. → S_1/S_2 CI TS (S_1)	3759	38017	40651	44203	22856	36239	37398	38409	41613	43547	44748
S_0 Min. → S_1/S_2 CI TS (S_2)	4815	43713	48224	46606	31525	35775	37483	38992	43399	44946	46283
S_0 Min. → X_5/X_6 CI TS (S_1)	2522	37499	42126	42525	21160	35884	36693	39302	40360	42923	46674

Table S4. Energies of the ground and excited states (first three singlet and first seven triplet) of *p*-HMC optimised during transition state searches between geometries found between s-*cis* OH-*syn* conformers (Table S2) using ωB97X (TD)DFT/6-311G(d,p) as implemented in ORCA 5.0.1[2–6]. Energies are relative to the ground state at the Franck-Condon point (S_0 minimum) for the s-*cis* OH-*anti* conformer (Table S1).

	Relative Energies (cm^{-1})										
**Optimised Geometry**	S_0	S_1	S_2	S_3	T_1	T_2	T_3	T_4	T_5	T_6	T_7
S_0 Min. → S_1/S_2 CI TS (S_1)	2582	37378	42190	42620	21091	35710	36893	39371	40780	42800	46773
S_1/S_2 CI → S_1/T_5 MECP TS (S_1)	10917	37846	46805	51823	30142	34748	42759	46638	48227	49572	49820
S_0 Min. → S_1 Min. TS (S_1)	2918	37673	42913	43144	21450	36045	37240	40324	41111	43131	46580

Table S5. Energies of the ground and excited states (first three singlet and first seven triplet) of *p*-HMC optimised during transition state searches between geometries found between s-*cis* OH-*syn* (Table S2) and s-*cis* OH-*anti* (Table S1) conformers using ωB97X (TD)DFT/6-311G(d,p) as implemented in ORCA 5.0.1[2–6]. Energies are relative to the ground state at the Franck-Condon point (S_0 minimum) for the s-*cis* OH-*anti* conformer (Table S1).

	Relative Energies (cm^{-1})										
**Optimised Geometry**	S_0	S_1	S_2	S_3	T_1	T_2	T_3	T_4	T_5	T_6	T_7
S_0 Min. → S_1/S_2 CI TS (S_1)	2582	37378	42190	42620	21091	35710	36893	39371	40780	42800	46773
S_1/S_2 CI → S_1/T_5 MECP TS (S_1)	10917	37846	46805	51823	30142	34748	42759	46638	48227	49572	49820
S_0 Min. → S_1 Min. TS (S_1)	2918	37673	42913	43144	21450	36045	37240	40324	41111	43131	46580

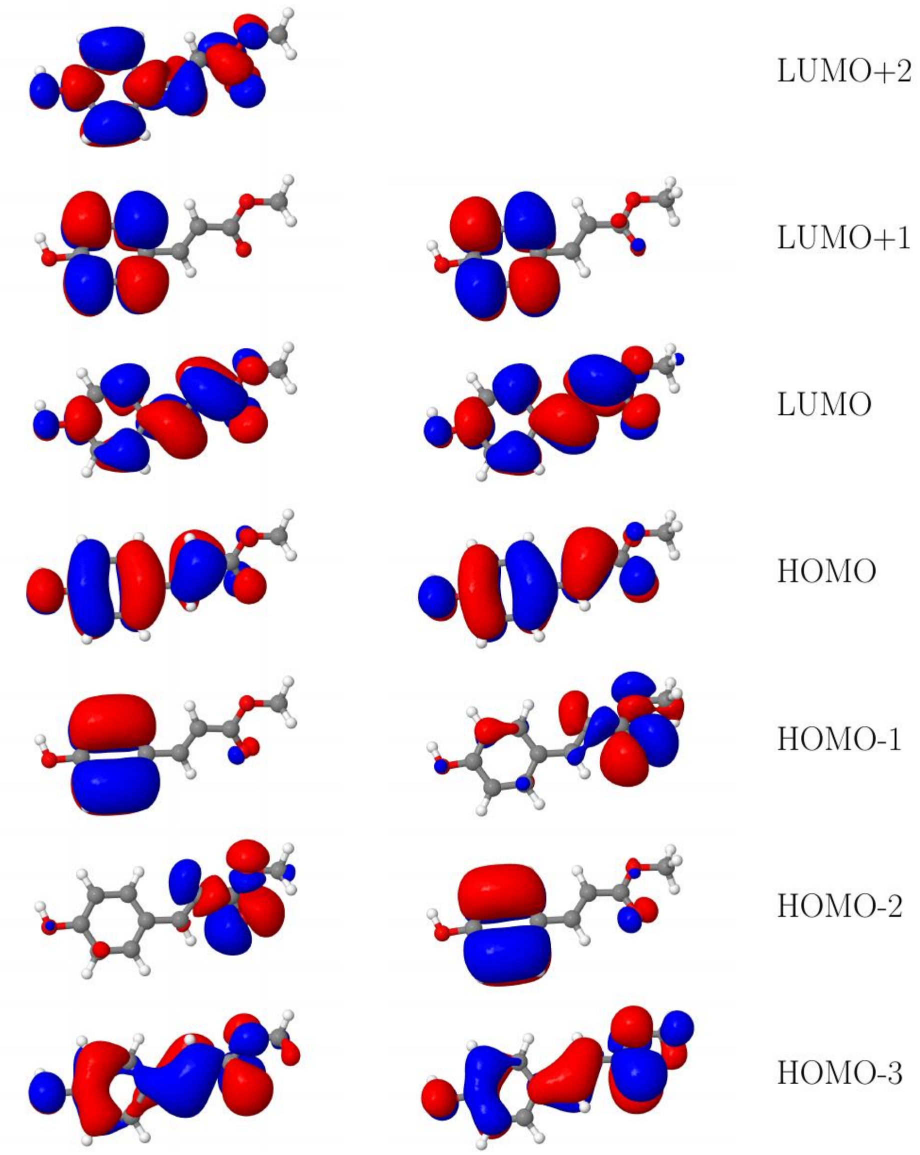

Figure S6. Molecular orbitals at the *syn*-conformer minimum energy crossing points (MECPs) labelled X_5/X_6 (S_1/T_5) and X_6/X_7 (S_2/T_5) in Figure 6 in the main text. On the left-hand side are those at the X_5/X_6 crossing point: the S_1 state is characterised by excitations from HOMO-2 to the virtual orbitals (hence its nπ* character) and the T_5 state is characterised by excitations from HOMO-3, HOMO-1 and HOMO to the virtual orbitals (hence its ππ* character). The orbitals on the right are those at the X_6/X_7 MECP: the S_2 state is characterised by 68% excitation from the HOMO to the LUMO (ππ*) with 20% from HOMO-1 to the LUMO (nπ*), whilst the T_5 state is characterised by excitations from HOMO-3 and HOMO-2 to the virtual orbitals (ππ*).

References

(1) Dobber, M. R.; Buma, W. J.; de Lange, C. A. Two-Color Picosecond Time-Resolved (2 + 1') Resonance-Enhanced Multiphoton Ionization Photoelectron Spectroscopy on the B1E'' and C' 1A1' States of Ammonia. *J. Phys. Chem.* **1995**, *99*, 1671–1685.

(2) Neese, F. The ORCA Program System. *Wiley Interdiscip. Rev. Comput. Mol. Sci.* **2012**, *2*, 73–78.

(3) Ekström, U.; Visscher, L.; Bast, R.; Thorvaldsen, A. J.; Ruud, K. Arbitrary-Order Density Functional Response Theory from Automatic Differentiation. *J. Chem. Theory Comput.* **2010**, *6*, 1971–1980.

(4) Weigend, F. Accurate Coulomb-Fitting Basis Sets for H to Rn. *Phys. Chem. Chem. Phys.* **2006**, *8*, 1057–1065.

(5) Krishnan, R.; Binkley, J. S.; Seeger, R.; Pople, J. A. Self-Consistent Molecular Orbital Methods. XX. A Basis Set for Correlated Wave Functions. *J. Chem. Phys.* **1980**, *72*, 650–654.

(6) Frisch, M. J.; Pople J. A.; Binkley, J. S. Self-Consistent Molecular Orbital Methods 25. Supplementary Functions for Gaussian Basis Sets. *J. Chem. Phys.* **1984**, *80*, 3265–3269.
